# CD8α Dendritic Cells Drive Establishment of HSV-1 Latency

**DOI:** 10.1371/journal.pone.0093444

**Published:** 2014-04-02

**Authors:** Kevin R. Mott, Sariah J. Allen, Mandana Zandian, Bindu Konda, Behrooz G. Sharifi, Clinton Jones, Steven L. Wechsler, Terrence Town, Homayon Ghiasi

**Affiliations:** 1 Center for Neurobiology and Vaccine Development, Ophthalmology Research, Department of Surgery, Cedars-Sinai Burns & Allen Research Institute, Los Angeles, California, United States of America; 2 Departments of Neurosurgery and Biomedical Sciences, Maxine Dunitz Neurosurgical Institute, Cedars-Sinai Medical Center, Los Angeles, California, United States of America; 3 Division of Cardiology, Department of Medicine, Cedars-Sinai Medical Center, Los Angeles, California, United States of America; 4 School of Veterinary Medicine and Biomedical Sciences, Nebraska Center for Virology, University of Nebraska, Lincoln, Nebraska, United States of America; 5 Gavin Herbert Eye Institute, The Department of Ophthalmology, The Department of Microbiology and Molecular Genetics, and the Center for Virus Research, University of California Irvine, School of Medicine, Irvine, California, United States of America; 6 Zilkha Neurogenetic Institute, Department of Physiology and Biophysics, Keck School of Medicine of the University of Southern California, Los Angeles, California, United States of America; Wayne State University School of Medicine, United States of America

## Abstract

It is generally accepted that CD8 T cells play the key role to maintain HSV-1 latency in trigeminal ganglia of ocularly infected mice. Yet, comparably little is known about the role of innate immunity in establishment of viral latency. In the current study, we investigated whether CD8α DCs impact HSV-1 latency by examining latency in the trigeminal ganglia (TG) of wild-type (WT) C57BL/6 versus CD8α^−/−^ (lack functional CD8 T cells and CD8α^+^ DCs), CD8β^−/−^ (have functional CD8α^+^ T cells and CD8α^+^ DCs), and β2m^−/−^ (lack functional CD8 T cells but have CD8α^+^ DCs) mice as well as BXH2 (have functional CD8 T cells but lack CD8α^+^ DCs) versus WT C3H (have functional CD8α T cells and CD8α^+^ DCs) mice. We also determined whether the phenotype of CD8α^−/−^ and BXH2 mice could be restored to that of WT mice by adoptive transfer of WT CD8^+^ T cells or bone marrow (BM) derived CD8α^+^ DCs. Our results clearly demonstrate that CD8α DCs, rather than CD8 T cells, are responsible for enhanced viral latency and recurrences.

## Introduction

HSV-1 infections are among the most frequent infections in the U.S. In addition to eye disease, HSV-1 can cause recurring orolabial lesions, pharyngitis, genital lesions, and less commonly, encephalitis [1]–[3]. It is estimated that 70–90% of the adult population in the U.S. has antibodies to HSV-1 and/or HSV-2, with about 25% showing clinical symptoms [4]–[6]. At various times throughout the life of the latently-infected individual, the virus may reactivate, travel back to the original site of infection and cause recurrent disease [2], [3]. Episodic recurrences−not primary infection−are the causative mechanism of corneal scarring (CS), which is also broadly referred to as herpes stromal keratitis (HSK) [7]–[10]. Despite the seriousness of recurrent ocular herpes, no drug has been FDA approved that prevents ocular recurrences. There is a critical need to understand the mechanism(s) behind HSV-1 latency so that effective methods for prevention and control of serious HSV-1-induced ocular syndromes can be devised.

Previously, it was shown that CD8 T cells infiltrate trigeminal ganglia (TG) at the time of HSV-1 latency establishment, where they have been hypothesized to inhibit reactivation from latency [11]. During latency, a subset of CD8 T cells remain in direct contact with infected neurons [12]. These cells can block HSV-1 reactivation from latency in *ex vivo* cultures of TG from latently-infected mice [11], [12]. We previously demonstrated that mice latently infected with wild-type (WT) HSV-1 have increased Latency-Associated Transcript (LAT) mRNA, and both increased CD8 and greater abundance of PD-1 mRNAs in their TGs, relative to mice latently infected with LAT deficient virus [13], [14]. More recently, our group found significantly decreased HSV-1 latency in PD-1^−/−^ and PD-L1^−/−^ mice compared to WT or PD-L2^−/−^ mice [13]. Further, we reported that mice depleted of their DCs by diphtheria toxin had lower levels of latency than mock-depleted control counterparts, and that myeloid DCs regulated this process [15]. Collectively, these studies suggest that DCs have a previously unappreciated function to modulate HSV-1 latency. While the potential role of LAT in this process is unclear, greater LAT production may result in increased DC infectivity, less anti-viral responses, and thus greater propensity for latency.

In this report, we examined latency in TG of WT versus CD8α^−/−^ and BXH2 mice, both of which do not have functional CD8α^+^ DCs [16], [17]; while CD8α^−/−^ mice lack and BXH2 mice have CD8 T cells. Additionally, β2m^−/−^ (lack functional CD8 T cells) and CD8β^−/−^ (have functional CD8 T cells) mice that have CD8α^+^ DCs were utilized. We determined if DCs from these mice behave differently following infection with LAT(+) versus LAT(−) viruses, and whether the phenotype of CD8α^−/−^ mice could be restored to that of WT mice by adoptive transfers of WT CD8^+^ T cells or bone marrow (BM) derived CD8α DCs. These studies point to a key role for CD8α^+^ DCs in establishment and maintenance of HSV-1 latency.

## Materials and Methods

### Ethics Statement

All animal procedures were performed in strict accordance with the Association for Research in Vision and Ophthalmology Statement for the Use of Animals in Ophthalmic and Vision Research and the NIH *Guide for the Care and Use of Laboratory Animals* (ISBN 0-309-05377-3). Animal research protocol was approved by the Institutional Animal Care and Use Committee of Cedars-Sinai Medical Center.

### Virus and Mice

Plaque-purified virulent HSV-1 strains McKrae and avirulent KOS were grown in rabbit skin (RS) cell monolayers in minimal essential medium (MEM) containing 5% fetal calf serum (FCS), as described previously [18]. RS (rabbit skin) cells (from Steven L Wechsler) was described previously [19].

WT C57BL/6, C57BL/6-CD8α**^−/−^**, C57BL/6-β_2_m**^−/−^**, C57BL/6-DTR, C57BL/6-GFP, BXH2/TyJ and C3H/HEJ mice were purchased from Jackson Laboratories. C57BL/6-CD8β**^−/−^** mice have been reported previously [20] and were bred in-house.

### Ocular Infection

Mice on the C57BL/6 background were infected ocularly with 2×10^5^ PFU per eye of HSV-1 strain McKrae, while BXH2/TyJ and C3H/HEJ mice were ocularly infected with 2×10^3^ of McKrae or 2×10^5^ PFU/eye of avirulent HSV-1 strain KOS due to their susceptibility to infection with higher PFU of HSV-1 strain McKrae. Each virus was suspended in 2 μl of tissue culture medium and administered as an eye drop without prior corneal scarification. Survival data in different mouse strains following infection with various PFU of HSV-1 strain McKrae are shown in Supplementary Table 1.

### DC Culture

Six week-old mice were used as a source of bone marrow (BM) for the generation of mouse DCs in culture. BM cells were isolated by flushing femurs with PBS. Pelleted cells were briefly resuspended in red blood lysing buffer (Sigma) to lyse red blood cells and stabilized by adding complete medium (RPMI 1640, 10% fetal bovine serum, 100 U/ml penicillin, 100 μg/ml streptomycin, 2 mM L-glutamine). The cells were centrifuged and resuspended in complete medium supplemented with GM-CSF (100 ng/ml; Peprotech, NJ) to enhance replication of DCs [21], [22]. Afterwards, cells were plated in non-tissue culture plastic Petri dishes (1 bone per 10 cm dish) for 5 days at 37°C with CO_2_.

### Infection of DCs

Monolayers of DCs from WT, β_2_m**^−/−^**, CD8α**^−/−^**, or CD8β**^−/−^** were infected with 1 PFU/cell of HSV-1 strains McKrae. One hr after infection at 37°C, virus was removed and the infected cells were washed three times with fresh medium and growth medium was replaced. At 24 hr post infection, infected DCs monolayers were harvested and the presence of gB transcript was determined by qRT-PCR.

### Adoptive Transfer of BM or CD8α DCs to Recipient Mice

BM from C57BL/6-GFP mice was isolated and each recipient CD8α^−/−^ mouse received BM from one donor mouse. We compared Intravenous (IV) and Intraperitoneal injection (IP) routes of BM transfer and found that IV transfer was more efficient than IP; therefore, all the experiments described here are based on IV transfer of BM. Two weeks post-transfer, the CD8α^−/−^ recipient mice were ocularly infected with HSV-1 strain McKrae. TG, BM, spleen, and thymus from recipient mice were isolated on the same day as ocular infection, and 14 and 28 days post ocular infection. Tissues were used for flow cytometry and IHC. TG from infected mice were also isolated for detection of LAT RNA and gB DNA on day 28 PI. For transfer to BXH2 mice, cultured DCs from C3H/HEJ mice were grown as above, harvested on day 5, and CD8α DCs were isolated by two cycles of positive selection per the manufacturer’s protocol (Miltenyi Biotec, Auburn CA). The purity of isolated CD8α DCs was more than 97% as confirmed by FACS analysis using anti-CD11c/anti-CD8α antibodies. Each recipient BXH2 mouse received CD8α DCs from one donor mouse and two weeks post-transfer, the recipient mice were ocularly infected with HSV-1 strain KOS. TG from infected mice were also isolated for detection of LAT RNA on day 28 PI.

### Adoptive Transfer of CD8 T Cells

Donor WT C57BL/6-GFP mouse spleens were pooled, and single-cell suspensions were prepared as described previously [23]. CD8 T cells were isolated using magnetic beads as described by the manufacturer (Miltenyi Biotec). After two cycles of purification, the purity of isolated CD8 T cells was 97% as confirmed by FACS analysis using anti-CD3/anti-CD8α antibodies. Each recipient mouse was injected once with CD8^+^ T cells from one donor mouse in 300 μl of MEM intraperitoneally. The control mice received 300 μl MEM alone. We compared IV and IP routes of CD8^+^ T cells transfer and detected no differences; therefore, all the experiments described here are based on IP transfer of CD8 T cells. The recipient and control mice were infected ocularly with strain McKrae 2 weeks after transfer of the CD8^+^ T cells. Presence of CD8 T cells in the spleen, thymus, BM, and TG of recipient mice was determined by IHC and flow cytometry before ocular infection, and at weeks 2 and 4 PI. TG from infected mice were also isolated for detection of LAT RNA and gB DNA on day 28 PI.

### Confocal Microscopy and Image Analysis

DCs isolated from different strains of mice were grown on Lab-Tex chamber slides (Sigma-Alderich) as we described previously [24]. Briefly, cells were fixed by incubating slides in methanol for 10 min followed by acetone for 5 min at −20°C. Afterwards, slides were rinsed three times for 5 min each at ambient temperature in PBS containing 0.05% v/v Tween-20 (PBS-T). Slides were then blocked for 30 min at ambient temperature in PBS-T containing 1% w/v BSA. Rat anti-CD8α (clone 53–6.7, eBioscience, San Diego, CA), rat anti-CD4 (clone Gk1.5, eBioscience), rat anti-CD8β (clone YTS156.7.7, BioLegend), and hamster anti-CD11c (clone HL3, BD Biosciences) were used for IHC. Immunostaining was done using CD11c/CD4, CD11c/CD8α, or CD11c/CD8β antibody combinations and staining for 1 h at 25°C. After three rinses for 5 min each in PBS, slides were incubated for 1 h at 25°C with secondary antibodies labeled with anti-hamster FITC or anti-Rat TRITC (Invitrogen). Slides were washed three times with PBS, air-dried and mounted with Prolong Gold DAPI mounting medium (Invitrogen). Images were captured at 1024×1024 pixels (original magnification = 20X) in independent fluorescence channels using a Nikon C1 eclipse inverted confocal microscope.

### DNA Extraction and PCR Analysis for HSV-1 Genomic DNA

DNA was isolated from homogenized individual TG using the commercially available DNeasy Blood &Tissue Kit (Qiagen, Stanford, CA) according to the manufacturer’s instructions. PCR analyses were done using gB specific primers (Forward - 5′-AACGCGACGCACATCAAG-3′; Reverse - 5′-CTGGTACGCGATCAGAAAGC-3′; and Probe - 5′-FAM-CAGCCGCAGTACTACC-3′). The amplicon length for this primer set was 72 bp. Relative copy numbers for gB DNA were calculated using standard curves generated from the plasmid pAc-gB1. In all experiments, GAPDH was used for normalization of transcripts.

### RNA Extraction, cDNA Synthesis, and TaqMan RT-PCR

RNA was extracted from latent TG or infected DCs as we have described previously [24]–[26]. Following RNA extraction, 1 μg of total RNA was reverse-transcribed using random hexamer primers and Murine Leukemia Virus (MuLV) Reverse Transcriptase from the High Capacity cDNA Reverse Transcription Kit (Applied Biosystems, Foster City, CA), in accordance with the manufacturer’s recommendations. The levels of various transcripts were evaluated using commercially available TaqMan Gene Expression Assays (Applied Biosystems) with optimized primer and probe concentrations. Primer-probe sets consisted of two unlabeled PCR primers and the FAM^™^ dye-labeled TaqMan MGB probe formulated into a single mixture. Additionally, all cellular amplicons included an intron-exon junction to eliminate signal from genomic DNA contamination. The HSV-1 gB and LAT primers and probe used were as follows: 1) gB: forward primer, 5′-AACGCGACGCACATCAAG-3′, reverse primer, 5′-CTGGTACGCGATCAGAAAGC-3′; and probe, 5′-FAM-CAGCCGCAGTACTACC-3′ – Amplicon length = 72 bp; and 2)LAT: forward primer 5′-GGGTGGGCTCGTGTTACAG-3′; reverse primer, 5′-GGACGGGTAAGTAACAGAGTCTCTA-3′; and probe, 5′- FAM-ACACCAGCCCGTTCTTT-3′– Amplicon Length = 81 bp, corresponding to LAT nts 119553–119634. As an internal control, a set of GAPDH primers from Applied Biosystems (ASSAY I.D. m999999.15_G1 - Amplicon Length = 107 bp) was used.

Quantitative real-time PCR (qRT-PCR) was performed using an ABI PRISM 7900HT Sequence Detection System (Applied Biosystems, Foster City, CA) in 384-well plates as we described previously [25], [26]. Real-time PCR was performed in triplicate for each tissue sample. The threshold cycle (Ct) values, which represents the PCR cycle at which there is a noticeable increase in the reporter fluorescence above baseline, were determined using SDS 2.2 Software. GAPDH transcript was used for normalization purposes.

### Statistical Analysis

Student’s t test and chi-squared tests were performed using the computer program Instat (GraphPad, San Diego). Results were considered statistically significant when the *P* value was <0.05.

## Results

### Effect of CD8 T Cells on the Establishment of Latency in Latently Infected Mice

**Figure 1:**
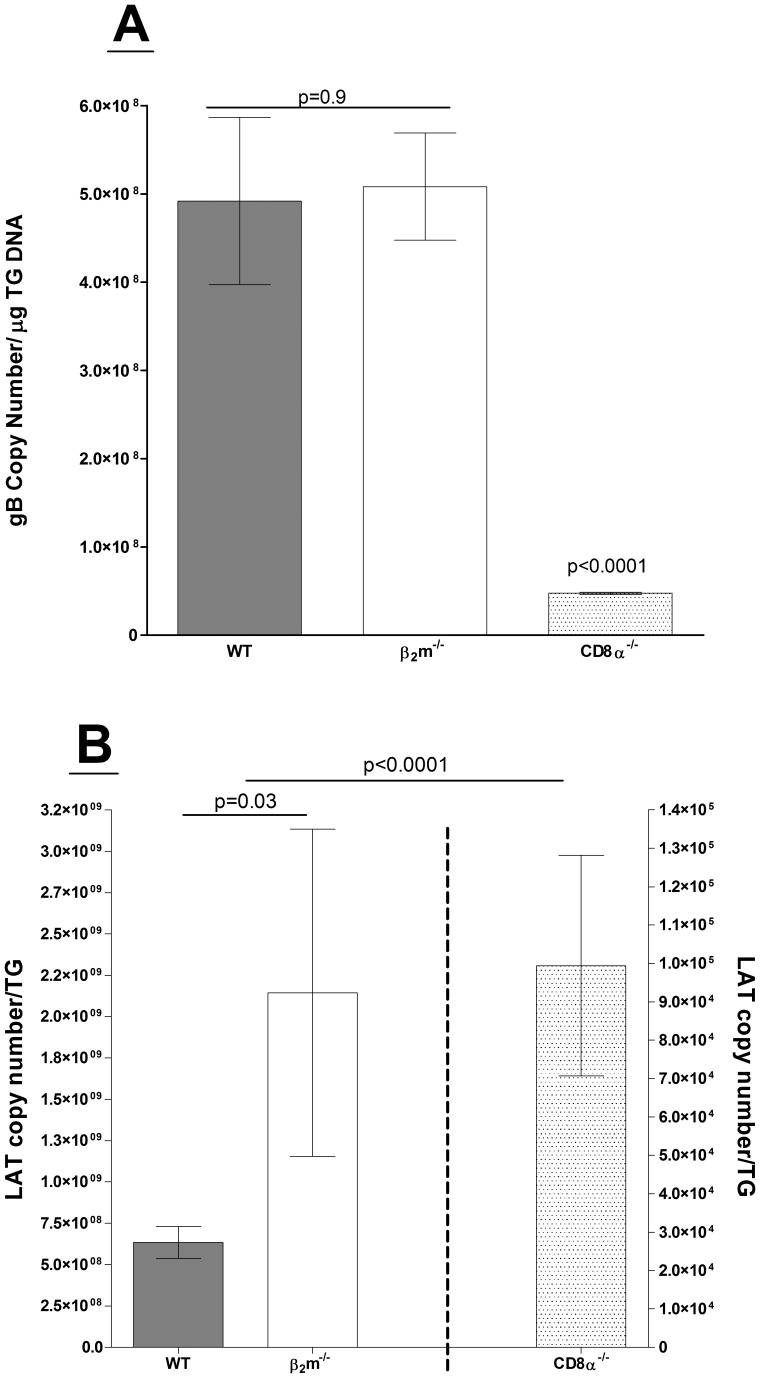
Quantitation of gB DNA and LAT RNA in trigeminal ganglia of HSV-1 latently-infected mice. Wild-type (WT) C57BL/6, β_2_m^−/−^, or CD8α^−/−^ mice were ocularly-infected with HSV-1 strain McKrae (LAT(+)). On day 28 PI, TG were harvested from latently infected mice. Quantitative PCR and RT-PCR was performed on each individual mouse TG. In each experiment, an estimated relative copy number of the HSV-1 gB (for viral DNA) and LAT (for viral RNA) were calculated using standard curves generated from pGem-gB1 and pGem5317, respectively. Briefly, DNA template was serially diluted 10-fold such that 5 μl contained from 10^3^ to 10^11^ copies of gB, then subjected to TaqMan PCR with the same set of primers. By comparing the normalized threshold cycle of each sample to the threshold cycle of the standard, the copy number for each reaction was determined. GAPDH expression was used to normalize the relative expression of viral (gB) DNA and LAT RNA in the TG. Each data point represents the mean ± SEM from 20 TGs for WT and 18 TGs for β_2_m^−/−^ or CD8α^−/−^ mice from two separate experiments. Panels: A) gB DNA; and B) LAT RNA (the Y-scale for LAT in CD8α^−/−^ mice TG is different than the Y-scale for WT and β_2_m^−/−^ mice).

It was now generally accepted that CD8 T cells play a dominant role to maintain HSV-1 latency [11]. We ocularly infected WT C57BL/6, C57BL/6-β_2_m^−/−^, and C57BL/6-CD8α^−/−^ mice with 2×10^5^ PFU/eye of WT HSV-1 strain McKrae. C57BL/6-CD8α^−/−^ mice are devoid of CD8^+^ T cells, and C57BL/6-β_2_m^−/−^ mice lack functional CD8^+^ T cells due to the absence of major histocompatibility complex I (MHC-I). Individual TG from surviving mice were isolated on day 28 post-infection (PI), and total DNA was prepared. TaqMan PCR was performed as described in Materials and Methods to quantitate viral gB DNA as a measure of HSV-1 genome copies and hence, latency. The combined data from two separate experiments are shown in Figure 1.

Surprisingly, the amount of viral DNA during latency in WT mice was similar to that of β_2_m^−/−^ mice (Figure 1A; WT vs. β_2_m^−/−^; p = 0.9). This suggests that lack of MHC-I and functional classical CD8 T cells does not impact viral latency but does not rule out the involvement of non-classical T cells in latency as was reported for gamma-herpesvirus 68 [27]. By contrast, gB copy number in TG of CD8α^−/−^ mice was reduced at least 6-fold compared to WT and β_2_m^−/−^ mice (Figure 1A, P<0.0001 for each comparison). These data demonstrate that the phenotype of CD8α^−/−^ mice significantly differs from β_2_m^−/−^ or WT mice with regards to latency.

During HSV-1 neuronal latency, only the LAT transcript is consistently expressed at high levels [28], [29]. We next sought to confirm our gB DNA latency results by quantitating LAT expression levels. On day 28, TG from surviving mice were harvested and LAT transcript was determined by TaqMan RT-PCR from total RNA isolated from individual TG. The amount of LAT RNA detected in the TG of β_2_m^−/−^ mice was significantly higher compared with WT mice (Figure 1B, *p*<.001). In agreement with the gB DNA results (Figure 1A), TG from CD8α^−/−^ mice had LAT copy numbers that were over 4 orders of magnitude lower than WT or β_2_m^−/−^ mice (Figure 1B, P<0.0001 for each comparison). These striking results demonstrate that absence of CD8α, but not β_2_m, reduces HSV-1 latency.

**Figure 2:**
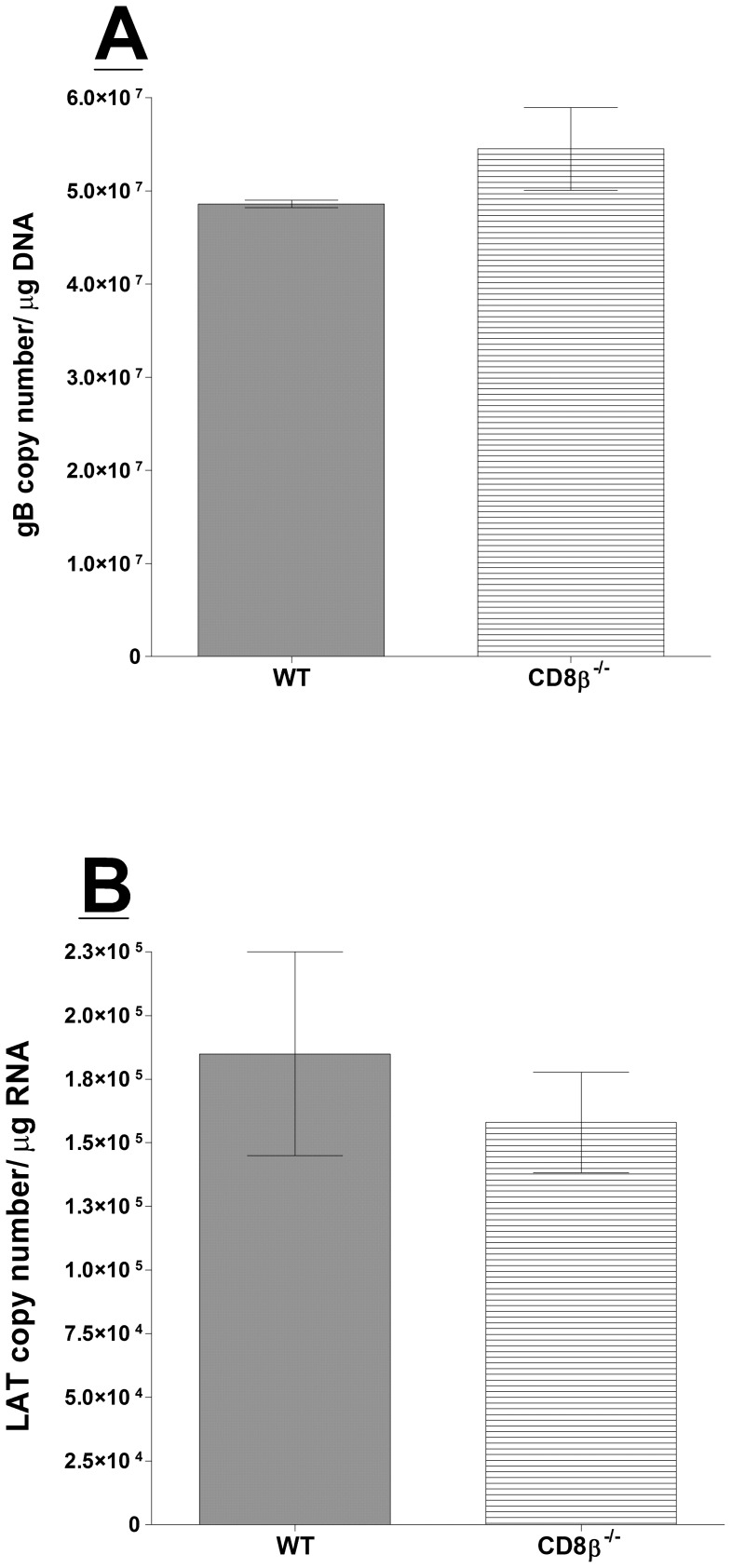
Quantitation of HSV-1 latency in CD8β^−/−^ infected mice. Wild-type (WT) C57BL/6 and CD8β^−/−^ mice were infected as described in Materials and Methods. Twenty-eight days PI, TGs from infected mice were harvested and quantitative PCR and RT-PCR were performed on each individual mouse TG. Each data point for gB DNA represents the mean ± SEM from 24 TGs. For LAT RNA, each data point represents 22 TGs for CD8β^−/−^ and 18 TGs for WT mice from two separate experiments. Panels: A) gB DNA; and B) LAT RNA.

CD8 consists of either αα homodimers or αβ heterodimers [30]. To follow-up from our CD8α^−/−^ mouse results, we next evaluated whether CD8β impacted viral latency using CD8β^−/−^ mice. CD8β^−/−^ and WT mice were ocularly infected as above, TG were harvested on day 28 PI, and TaqMan PCR and RT-PCR were performed as detailed above. Combined data from two separate experiments are shown in Figure 2. The amount of latency, as determined by viral DNA and LAT RNA, was similar between CD8β^−/−^ and WT mice (Figure 2A and B; p>0.05). Interestingly, absence of the CD8β chain did not affect latency. CD8β^−/−^ mice have attenuated T cell function but normal numbers of functional CD8α DCs [20]. These results raise the possibility that CD8α DCs−rather than CD8 T cells−play the primary role in HSV-1 latency establishment.

**Figure 3:**
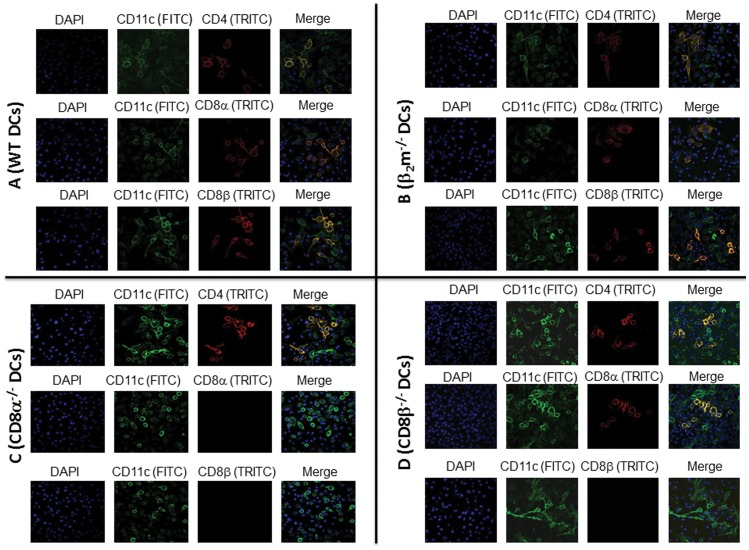
IHC of DCs isolated from different knockout of mice. BM-derived DCs from WT, β_2_m^−/−^, CD8α^−/−^, and CD8β^−/−^ mice were isolated and grown on Lab-Tex chamber slides. At 24 hr post culture, DCs were fixed, stained with anti-CD11c/anti-CD4, CD11c/anti-CD8α, or CD11c/anti-CD8β antibodies followed by incubation with relevant secondary antibody to each primary antibody as described in Materials and Methods. DAPI is shown as a nuclear counter-stain. A (WT DCs). Upper panels from left to right DAPI, CD11c (FITC), CD4 (TRITC), Merge; middle panels: from left to right DAPI, CD11c (FITC), CD8α (TRITC), Merge and bottom panels: from left to right DAPI, CD11c (FITC), CD8β (TRITC), Merge; B (β_2_m^−/−^ DCs). Upper panels from left to right DAPI, CD11c (FITC), CD4 (TRITC), Merge; middle panels: from left to right DAPI, CD11c (FITC), CD8α (TRITC), Merge, and bottom panels: from left to right DAPI, CD11c (FITC), CD8β (TRITC), Merge; C (CD8α^−/−^ DCs). Upper panels from left to right DAPI, CD11c (FITC), CD4 (TRITC), Merge; middle panels: from left to right DAPI, CD11c (FITC), CD8α (TRITC), Merge, and bottom panels: from left to right DAPI, CD11c (FITC), CD8β (TRITC), Merge; and D (CD8β^−/−^ DCs). Upper panels from left to right DAPI, CD11c (FITC), CD4 (TRITC), Merge; middle panels: from left to right DAPI, CD11c (FITC), CD8α (TRITC), Merge, and bottom panels: from left to right DAPI, CD11c (FITC), CD8β (TRITC), Merge.

Nonetheless, lower latency in CD8α^−/−^ compared with WT, β_2_m^−/−^, or CD8β^−/−^ mice could be owed to absence of CD8α T cells, CD8α DCs, or both. Mouse plasmacytoid DCs (pDCs) were previously thought to express CD8α, but not CD8β. However, it was recently shown that at least one DC subclass does express CD8β [31]. To evaluate expression profiles of CD8α and CD8β on DCs, BM-derived DCs from naive WT, β_2_m^−/−^, CD8α^−/−^, and CD8β^−/−^ mice were grown to confluency on chamber slides as described in Materials and Methods. Cells were stained with various combinations of CD11c, CD8α or CD8β antibodies, and CD11c/CD4 immunostaining was used as a positive control for each mouse strain. Immunohistochemistry (IHC) results are shown in Figure 3. CD4, CD8α, and CD8β were all detected on DCs isolated from WT and β_2_m^−/−^ mice (Figure 3A, WT DCs; 3B, β_2_m^−/−^ DCs). By contrast, CD8α or CD8β DCs were not detected on DCs isolated from CD8α^−/−^ mice, while CD4 DCs were present (Figure 3C, CD8α^−/−^ DCs). These results are consistent with a previous study showing that mouse CD8β cannot be expressed as a ββ homodimer in the absence of CD8α [32], [33]. In contrast, CD8β^−/−^ mice lacked CD8β DCs, while CD8α DCs were not affected (Figure 3D, CD8β^−/−^ DCs). Thus, these results allow us to group pDCs into CD8α^+^β^+^, CD8α^+^β^-^, and CD8α^-^β^-^. These data dovetail with a previous report showing distinct populations of pDCs according to their surface expression of CD8α or CD8β [31]. Taken together, these results suggest that lack of CD8α DCs alone or in combination with CD8 T cells reduces latency in CD8α^−/−^ mice compared with WT, β_2_m^−/−^, or CD8β^−/−^ mice.

### Adoptive Transfer of CD8α DCs, but not CD8 T Cells, Increases Latency in HSV-1-Infected CD8α^−/−^ Mice

**Figure 4:**
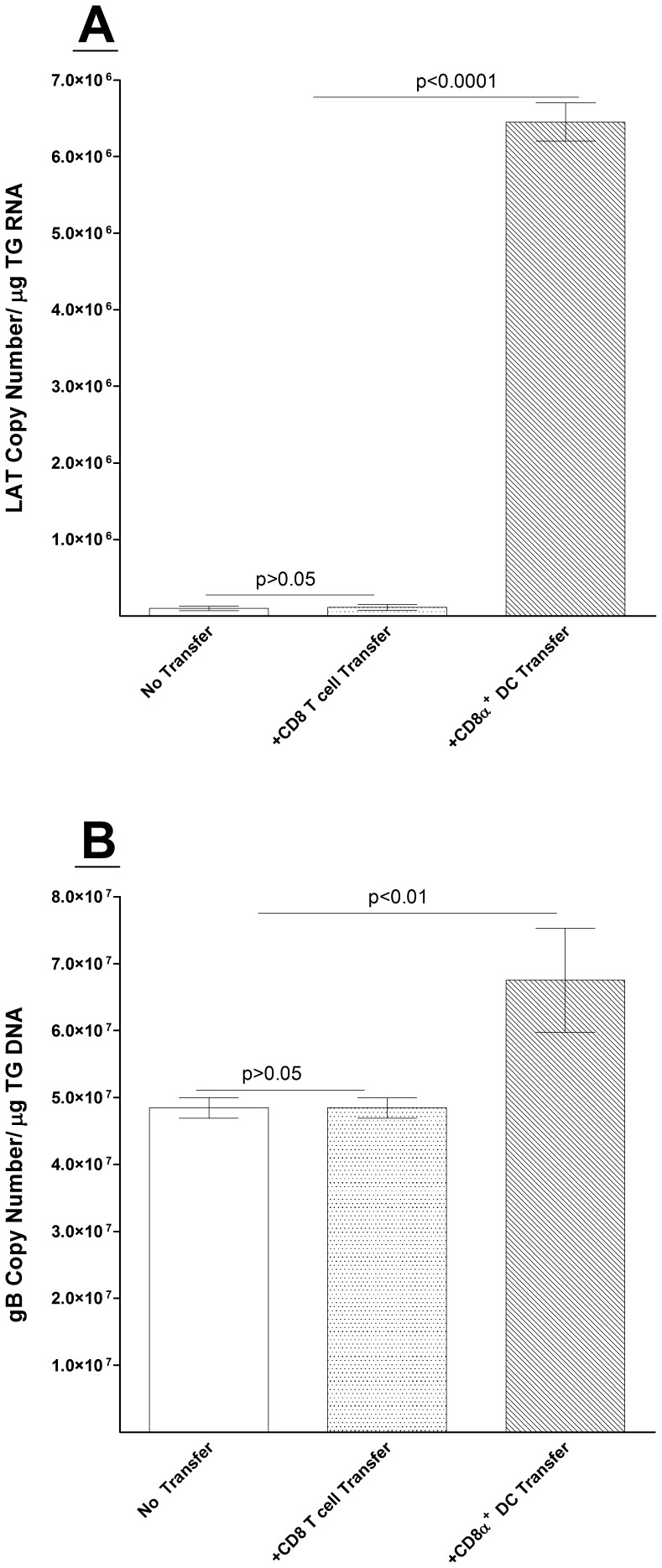
LAT and gB levels in latently-infected CD8α^−/−^ mice following adoptive transfer of bone marrow or CD3^+^CD8^+^ T cells. Each recipient CD8α^−/−^ mice received bone marrow (BM) or CD3^+^CD8^+^ T cells as described in Materials and Methods. Control mice did not receive adoptive transfer. Two weeks post-transfer, mice were ocularly infected as described in Materials and Methods. Quantitative RT-PCR and PCR was performed on RNA and DNA isolated from the TG of surviving mice to determine copy number of the LAT or gB transcripts, respectively. Each data point represents the mean ± SEM from 14 TGs. Panels: A) LAT RNA; and B) gB DNA.

In the above experiments using CD8α^−/−^ mice, we found significantly reduced viral latency compared with WT, β2m^−/−^, or CD8β^−/−^ mice. Therefore, it was of interest to determine if decreased latency was due to attenuated CD8α DCs or CD8 T cells. CD8α^−/−^ mice adoptively received either BM-derived CD8α DCs or T cells from WT GFP mice as described in Materials and Methods. Abundance of LAT RNA in mice that received BM-derived CD8α DCs was at least 6-fold higher than in mice that received CD8 T cells or no adoptive transfer (Figure 4A; BM DC transfer vs. no transfer or CD8 T cell transfer; p<0.0001). There was no difference in latency between mice that received CD8 T cells and no transfer control mice (Figure 4A, p>0.05). A similar pattern of results was observed when latency levels were judged by gB DNA (genomic DNA) levels (Figure 4B). Thus, adoptive transfer of BM-derived CD8α DCs significantly increased the levels of LAT and gB DNA in CD8α^−/−^ mice, while transfer of CD8 T cells had no effect. Figure S1A and S1B shows detection of GFP^+^CD8α^+^ T cells or GFP^+^CD8α^+^ DCs in the TG, BM, and spleen of recipient mice both before infection and at 14 and 28 days PI. Figure S1C shows the presence of GFP^+^CD8α^+^ T cells in the spleen, thymus and TG of ocularly infected mice on day 28 PI via immunohistochemistry. In summary, these results indicate that CD8α DCs, and not CD8 T cells, play an indispensable role in increasing HSV-1 latency.

### BXH2 Mice have Similar Viral Latency as CD8α^−/−^ Mice

**Figure 5:**
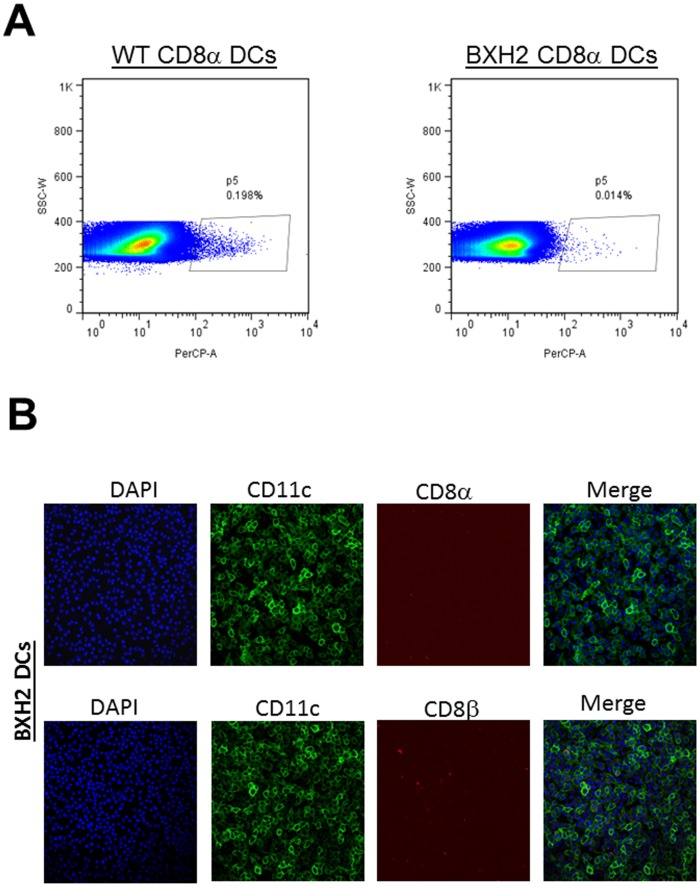
CD8α^+^ DCs in BXH2 mice. **A) FACS.** Subconfluent monolayers of DCs from WT C3H/HEJ and BXH2/TyJ mice were grown to confluency and stained with anti-CD8α antibody and analyzed by FACS. Left panel show expression of CD8α^+^ DCs in WT mice, while the right panel shows CD8α^+^ DCs in BXH2 mice. Number above each box indicates the percent of CD8α^+^ DCs per mouse strain. B) IHC. BM-derived DCs from BXH2 mice were isolated and grown on Lab-Tex chamber slides. At 24 hr post culture, DCs were fixed, stained with anti-CD11c/anti-CD8α or anti-CD11c/anti-CD8β antibodies followed by incubation with relevant secondary antibody to each primary antibody as described in Materials and Methods. DAPI is shown as a nuclear counter-stain. Upper panels from left to right DAPI, CD11c (FITC), CD8α (TRITC), and Merge; and bottom panels: from left to right DAPI, CD11c (FITC), CD8β (TRITC), and Merge.

**Figure 6:**
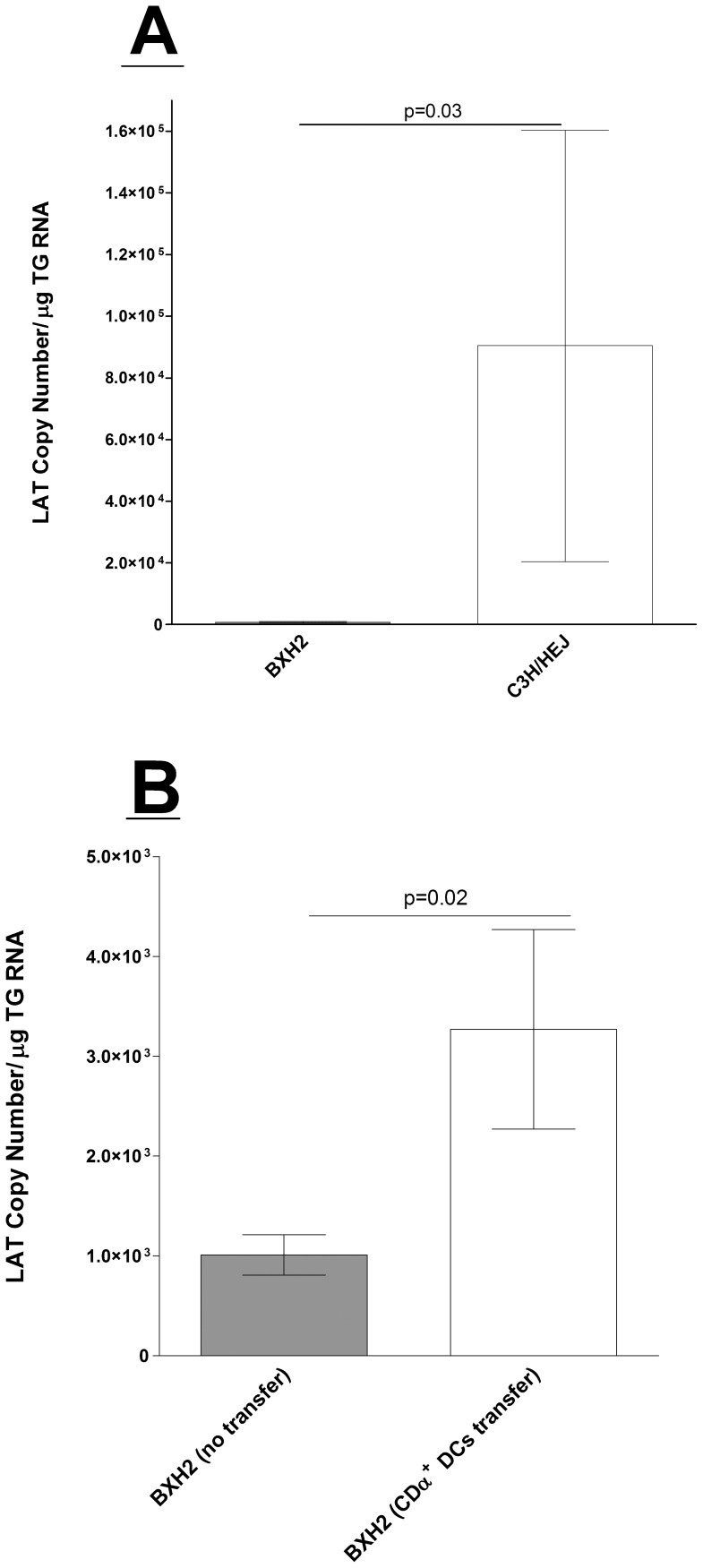
Quantitation of latency in BXH2 mice infected with HSV-1. A) Infection of mice with virulent HSV-1 strain McKrae. BXH2 or wild-type (WT) C3H/HEJ mice were infected with 2×10^3^ PFU/eye of virulent HSV-1 strain McKrae. Twenty eight days PI, TGs from infected mice were harvested and quantitative RT-PCR was performed on each individual mouse TG as described in Materials and Methods. Each data point represents the mean ± SEM from 18 or 12 TGs from BXH2 or WT mice from two separate experiments. B) Latency following adoptive transfer of CD8α DCs**.** Each recipient BXH2 mouse received BM-derived CD8α DCs from WT mice as described in Materials and Methods. A subset of BXH2 mice did not receive adoptive transfer, as a negative control. BM-derived recipient BXH2, untreated BXH2, and WT mice were ocularly infected with 2×10^5^ PFU/eye of avirulent HSV-1 strain KOS. Twenty-eight days PI, TG from infected mice were harvested and quantitative RT-PCR were performed on each individual mouse TG. Each data point represents the mean ± SEM from 18 TG from BM-derived recipient BXH2 or 16 TG from untreated BXH2 mice.

Previously, it was shown that interferon regulatory factor 8 (IRF-8) is essential for the development of CD8α myeloid DCs and pDCs [34]. More recently, a spontaneous point mutation was identified in the IRF-8 gene in the BXHBXH2 mouse [35]. This mutant has a similar phenotype as the IRF-8 knockout mouse, but without impaired pDC development and these mice are competent to produce type 1 IFNs [16]. As shown in Figure 5A, DCs isolated from BXH2 mice had no significant numbers of CD8α DCs compared with C3H/HEJ control mice. IHC results also confirmed the absence of CD8α as well as CD8β on these cells (Figure 5B). These results are similar to a previous report by Tailor et al showing that BXH2 mice do not have CD8α DCs but have intact CD8 T cell responses [16]. Thus, similar to CD8α^−/−^ mice, BXH2 mice lack CD8α DCs, but in contrast to CD8α^−/−^ mice, they have normal CD8 T cells. To determine if the absence of CD8α DCs, as opposed to CD8 T cells, contributed to higher latency in the CD8α^−/−^ mouse, we ocularly infected BXH2 mice with 2×10^3^ PFU/eye of HSV-1 strain McKrae. Control C3H/HEJ mice were similarly infected. The amount of LAT RNA detected in TG of BXH2 mice was significantly lower than control C3H/HEJ mice (Figure 6A, *p*<0.001).

To further confirm the role of CD8α DCs in establishment of viral latency, BXH2 mice adoptively received BM-derived CD8α DCs from WT C3H/HEJ mice as described in Materials and Methods. BXH2 untreated mice, BXH2 mice that received CD8α^+^ DCs, and control C3H/HEJ mice were ocularly infected with 2×10^5^ PFU/eye of avirulent HSV-1 strain KOS to reduce death of infected animals. Abundance of LAT RNA in mice that received BM-derived CD8α DCs was similar to control C3H/HEJ mice, and both were significantly higher when compared to untreated BXH2 mice (Figure 6B). Thus, adoptive transfer of CD8α DCs recovers the phenotype of the BXH2 mice. These results further bolster the notion that CD8α DCs−and not CD8 T cells−function to increase viral latency.

### HSV-1 Infectivity is Reduced in DCs Isolated from CD8α^−/−^ Mice

**Figure 7:**
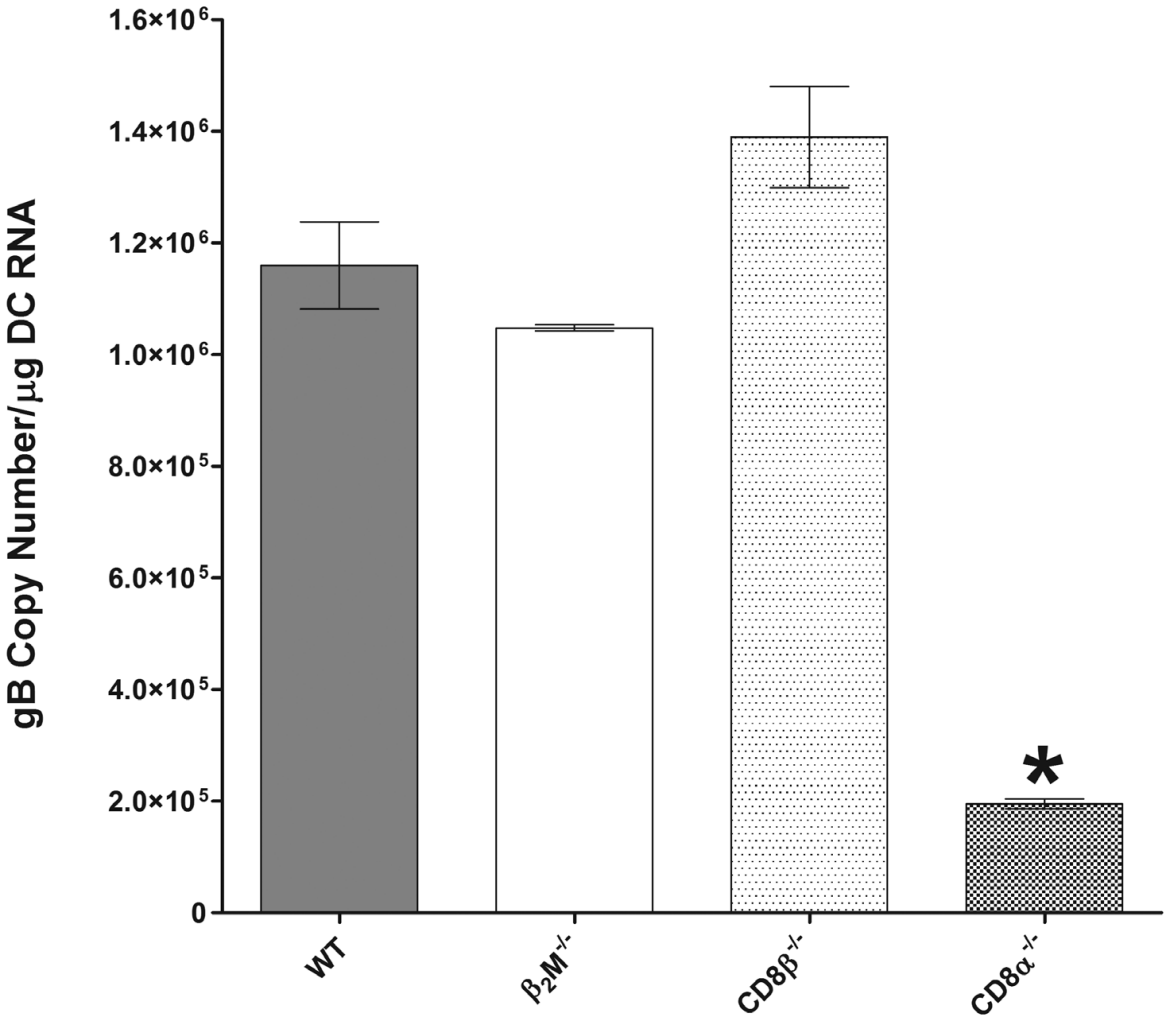
Replication of HSV-1 in bone marrow-derived DCs. Sub-confluent monolayers of DCs cultured from WT, β_2_m^−/−^, CD8β^−/−^, or CD8α^−/−^ mice were infected with 1 PFU/cell of HSV-1 strain McKrae for 24 h as described in Materials and Methods. RNA was isolated and the amount of gB transcript was determined by Taq-Man RT-PCR and normalized to GAPDH DNA. Each data point represents the mean ± SEM (n = 4).

The above experiments suggested that absence of CD8α DCs affected the level of latency *in vivo*. To determine if this effect also operated *in vitro*, DCs were isolated and cultured from naive WT, β_2_m^−/−^, CD8β^−/−^, or CD8α^−/−^ mice. DCs were then infected with 1 PFU/cell of HSV-1 strain McKrae, as described in Materials and Methods. At 24 h PI, RNA was isolated and gB transcript abundance was measured by qRT-PCR (Figure 7). Similar to our results with knockout mice, infected CD8α^−/−^ DCs had at least 10-fold less gB RNA than DCs from WT, β_2_m^−/−^, or CD8β^−/−^ mice (Figure 7, p<0.0001).

## Discussion

One of the hallmarks of HSV infection is the ability of the virus to establish latency in neurons of an infected host [36]–[38]. Once acquired, latent infections demonstrate a lifelong pattern of episodic recurrence, such that infected individuals serve as permanent carriers who are intermittently infectious [7]–[9]. The most efficient way to decrease latency and subsequent recurrent infections is to reduce establishment of latency, thus decreasing the probability of subsequent reactivations and recurrent eye disease. Previously, we showed that lymphoid-related DCs enhance HSV-1 latency in TG of infected mice and that immunization of mice with Flt3L, which increases lymphoid-related DCs, increases latent virus in the TG [15], [39].

DCs have been classified into several subsets based on their immunophenotype, resident location, and functional differences [40], [41]. CD8α DCs are specialized cells known for cross-presentation of antigens by MHC-I molecules and for their major role in priming cytotoxic CD8 T cell responses *in vivo* [42]–[45]. In addition, they have a high capacity to engulf apoptotic cells, which are used as a source of antigens for cross-priming [46]. Human blood DC antigen 3 (CD141, BDCA3) DCs are reported to be the homologue of mouse CD8α DCs [47]. To elucidate whether a relationship existed between CD8α DCs and HSV-1 latency, we utilized a multitude of gene deficient murine models. In this report, we show that increased viral latency is determined by CD8α DCs, but not CD8 T cells. Interestingly, we previously reported increased latency in CD4^−/−^ mice, indicating the importance of CD4 T cells in orchestrating immune responses against HSV-1 latency [39].

In contrast to CD8α^+^ DCs, CD8α^-^ DCs preferentially drive activation of CD4 T cells [48], can produce IFNs and IL-12 [49], acquire the ability to cross-present antigen [50], and induce CD4 cytotoxic regulatory T cells [51]–[54]. In addition, CD8α^-^ DCs are more effective than CD8α^+^ DCs at activating antigen-specific CD4 T cells [43], [55]. On the other hand, cross-presenting self-Ags is unique to CD8α^+^ DCs, and is either completely absent or less effective in other DC subsets [56]. However, the cross-presentation capacity of CD8α^+^ DCs is a double-edged sword, as it also increases their susceptibility to infection [57], [58]. Consequently, infected CD8α^+^ DCs may lose their function and/or block the APC function of other DC subtypes. This may lead to greater viral latency and loss of CD8 T cell function. Thus, lower latency in the absence of CD8α^+^ DCs, as found in this report, may be due to APC subtypes other than CD8α^+^ DCs being able to process antigen and induce a more effective antiviral response. Consistent with this notion, absence of CD8α^+^ DCs in mice enhances resistance to the intracellular bacterium *Listeria monocytogenes* [58]. These results are also consistent with reports on the negative role of certain DC populations in control of Vaccinia virus [59], HIV-1 [60], [61], and dengue virus [62]. In addition, the potential for autoimmunity induced by DCs, particularly in response to persistent viral infection, has been suggested by others [63].

Because of the critical role that DCs play in orchestrating immune responses, there is increasing interest in using signals that are known to activate DCs to stimulate and improve vaccine efficacy. However, the negative aspects of DCs have received far less attention than their positive roles, likely due to enthusiasm for their immunotherapeutic potential (reviewed in [64]). It was shown that Langerhans cells are negative regulators of the anti-*Leishmania* response in mice [65], similar to our results reported here for CD8α DCs and HSV-1. Additionally, Vaccinia virus abortively infects both mature and immature DCs and blocks their maturation; hence, T cell activation is impaired [59]. By inhibiting maturation pathways in DCs and inducing their death, Vaccinia virus can subvert the development of efficient antiviral T cell immunity. Similarly, we found here that DCs isolated from CD8α^−/−^ mice harbor fewer viruses than other populations of DCs. Consequently, HSV-1 infected DCs may directly, or via a negative feedback control mechanism, interfere with DC function, thus inhibiting induction of antiviral responses leading to even higher latency. Previously, it was shown that depletion of DCs during primary ocular infection reduces NK cells function and results in impaired clearance of HSV-1 from the eye of infected mice [66]. Maturation of DCs is required to induce potent immune responses, and HSV-1 infection elicits immune responses incapable of preventing or eradicating infection. Furthermore, we have previously shown that mature murine DCs can be infected by HSV-1, but the virus does not replicate in infected DCs [24]. Similarly, it was shown that following infection with HSV-1, immature DCs generate infectious viral particles, while mature DCs do not support virus production [67]. Finally, HSV-1 infection of human DCs results in functional impairment of the infected cells [68]–[70].

It has been shown that TG-resident CD8 T cells block HSV-1 reactivation from latency, and that these cells are necessary for maintaining viral latency [11], [12]. Additionally, CD8 T cells can actively suppress viral reactivation through release of IFN-γ [71]. It was reported that adding anti-CD8 antibody to cells isolated from TG of latently infected mice increases reactivation of latent virus [11]. Interestingly however, these studies all relied on CD8α antibody, and so their results might be reinterpreted as implicating CD8α DCs as opposed to CD8 T cells.

A key finding in our study is that increased latency is due to CD8α^+^ DCs. These results might also translate into neuroprotection, since increased latency usually means more neurons survive the primary infection. Along these lines, our published studies have shown that transfer of CD11c^+^CD8α^+^ cells significantly enhances latency in the TG of WT infected mice, whereas transfer of CD11c^+^CD8α^-^ cells reduces latency [39].

Overall, our results suggest that CD8α^+^ DCs, rather than CD8^+^ T cells, play a non-redundant role in increasing HSV-1 latency. Thus, we propose that infection of CD8α^+^ DCs by HSV-1 reduces the antiviral response, resulting in greater latency, which in turn leads to increased recruitment of CD8 T cells at the site of latent infection. Continuous exposure of these CD8 T cells to viral antigens results in increased PD-1/PD-L1 and T cell exhaustion as we reported recently [13]. Our results reveal a previously unappreciated role of innate immunity in maintaining HSV-1 latency.

### Conclusion

Previously, it was reported that CD8^+^ T cells infiltrate trigeminal ganglia (TG) at the time of latency establishment and contributing to increase of latency as well as inhibiting HSV-1 reactivation from latency. This has become the standard mechanism of HSV-1 latency-reactivation among herpes researchers. To determine factors that are contributing to increase of latency, we examined latency in TG of WT mice versus CD8α^−/−^ and BXH2 mice, both of which lack CD8α DCs but CD8α^−/−^ mice lack CD8^+^ T cells, while BXH2 mice have CD8^+^ T cells. Additionally, β2m^−/−^ and CD8β^−/−^ mice that have CD8α DCs were utilized. Overall, we report here that, in the absence of CD8α^+^ DCs, HSV-1 latency was significantly decreased *in vivo*. Thus, these studies point to a key role for CD8α^+^ DCs rather than CD8^+^ T cells in establishment and maintenance of HSV-1 latency in TG of infected mice.

## Supporting Information

Figure S1 **Efficiency of adoptive CD8α^+^ T cells or BM to recipient CD8α^−/−^ mice.** Naive CD8α^+^ T cells or BM were isolated from naive C57BL/6-GFP^+^ mice as described in Materials and Methods. Isolated CD8α^+^ T cells or BM were transferred IP or IV into recipient CD8α^−/−^ mice, respectively. Two weeks post transfer some of the recipient mice were infected ocularly with 2×10^5^ PFU/eye of WT HSV-1 strain McKrae. On day 14 before ocular infection and on days 14 and 28 PI some of the mice were euthanized and the presence of GFP^+^ cells in TG, BM, spleen and thymus were determined by FACS and IHC. A) Transfer of CD8α^+^GFP^+^ T cells to recipient mice. Presence of total GFP^+^ cells in TG, BM, and spleen of donor WT-GFP^+^ mice are shown as control in the left side under the WT-GFP column. Marked area inside each quadrant show presence of CD8α^+^GFP^+^ T cells in TG, BM, and spleen of recipient mice before and after infection; B) Transfer of BM-GFP^+^ cells to recipient mice. Marked area inside each quadrant show presence of GFP^+^ in TG, BM, and spleen of recipient mice before and after infection; and C) Detection of CD8α^+^GFP^+^ T cells in recipient mice. Presence of CD8α^+^GFP^+^ T cells in TG, thymus, and spleen of recipient mice were determined by IHC using anti-GFP-488 antibody to enhance the signal. DAPI is shown as a nuclear counter-stain. Spleen (upper panels): from left to right DAPI, anti-GFP-488, and Merge; Thymus (middle panels): from left to right DAPI, anti-GFP-488, and Merge; and TG (Bottom): from left to right DAPI, anti-GFP-488, and Merge.
(PPT)

Table S1 **Survival of different mouse strains following ocular infection.**
(DOCX)
