# Supplementary material for: CD8α Dendritic Cells Drive Establishment of HSV-1 Latency
Source: PLoS One. 2014 Apr 2;9(4):e93444. doi: 10.1371/journal.pone.0093444 (PMC3973679; doi:10.1371/journal.pone.0093444)
Supplement: Figure S1 — Efficiency of adoptive CD8α+ T cells or BM to recipient CD8α−/− mice. Naive CD8α+ T cells or BM were isolated from naive C57BL/6-GFP+ mice as described in Materials and Methods. Isolated CD8α+ T cells or BM were transferred IP or IV into recipient CD8α−/− mice, respectively. Two weeks post transfer some of the recipient mice were infected ocularly with 2×10^5^ PFU/eye of WT HSV-1 strain McKrae. On day 14 before ocular infection and on days 14 and 28 PI some of the mice were euthanized and the presence of GFP+ cells in TG, BM, spleen and thymus were determined by FACS and IHC. A) Transfer of CD8α+GFP+ T cells to recipient mice. Presence of total GFP+ cells in TG, BM, and spleen of donor WT-GFP+ mice are shown as control in the left side under the WT-GFP column. Marked area inside each quadrant show presence of CD8α+GFP+ T cells in TG, BM, and spleen of recipient mice before and after infection; B) Transfer of BM-GFP+ cells to recipient mice. Marked area inside each quadrant show presence of GFP+ in TG, BM, and spleen of recipient mice before and after infection; and C) Detection of CD8α+GFP+ T cells in recipient mice. Presence of CD8α+GFP+ T cells in TG, thymus, and spleen of recipient mice were determined by IHC using anti-GFP-488 antibody to enhance the signal. DAPI is shown as a nuclear counter-stain. Spleen (upper panels): from left to right DAPI, anti-GFP-488, and Merge; Thymus (middle panels): from left to right DAPI, anti-GFP-488, and Merge; and TG (Bottom): from left to right DAPI, anti-GFP-488, and Merge. (PPT) [file pone.0093444.s001.ppt]

## Slide 1
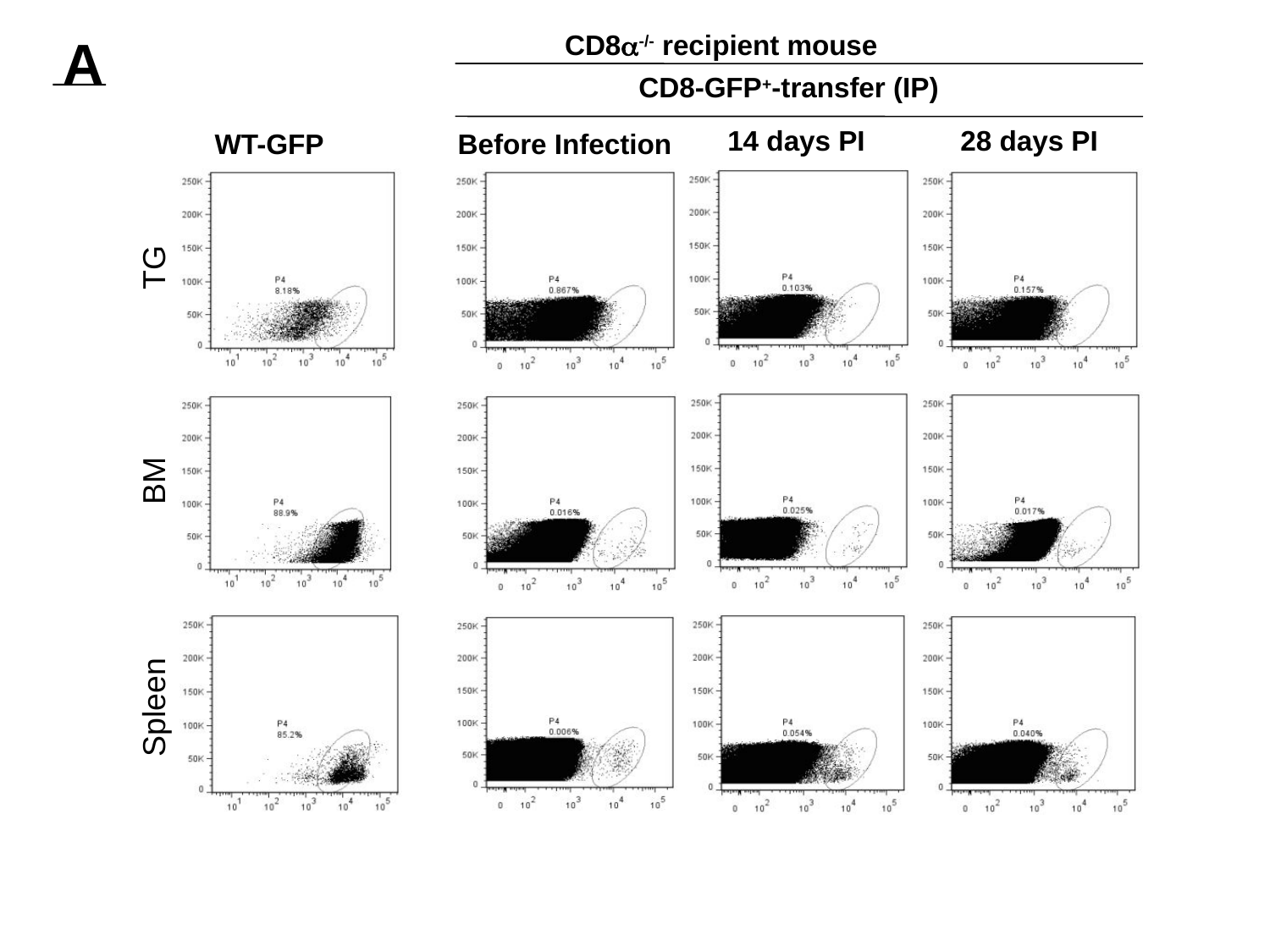

CD8α-/- recipient mouse
CD8-GFP+-transfer (IP)
14 days PI
28 days PI
Before Infection
A
WT-GFP
TG
BM
Spleen

## Slide 2
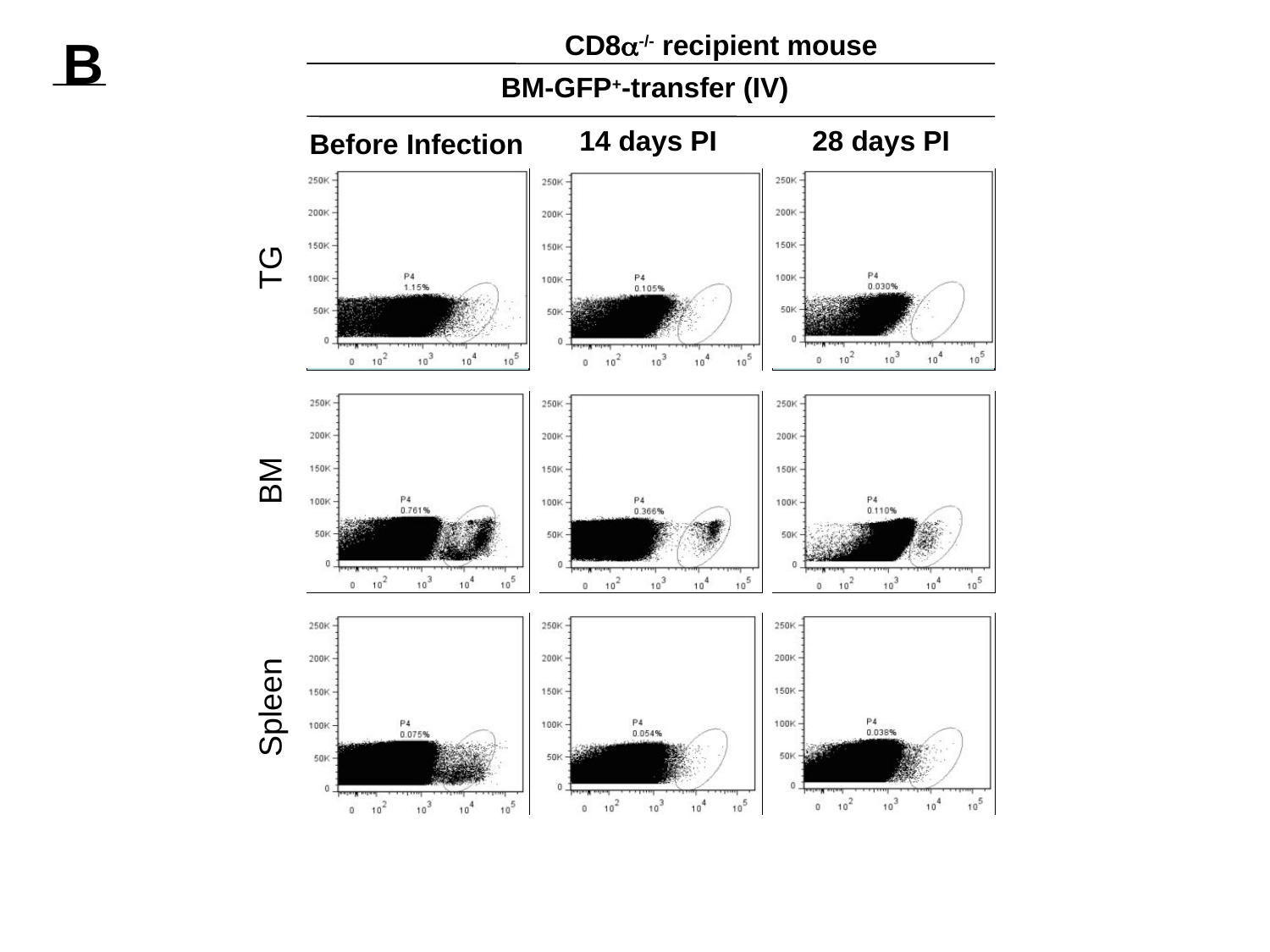

CD8α-/- recipient mouse
BM-GFP+-transfer (IV)
14 days PI
28 days PI
Before Infection
TG
BM
Spleen
B

## Slide 3
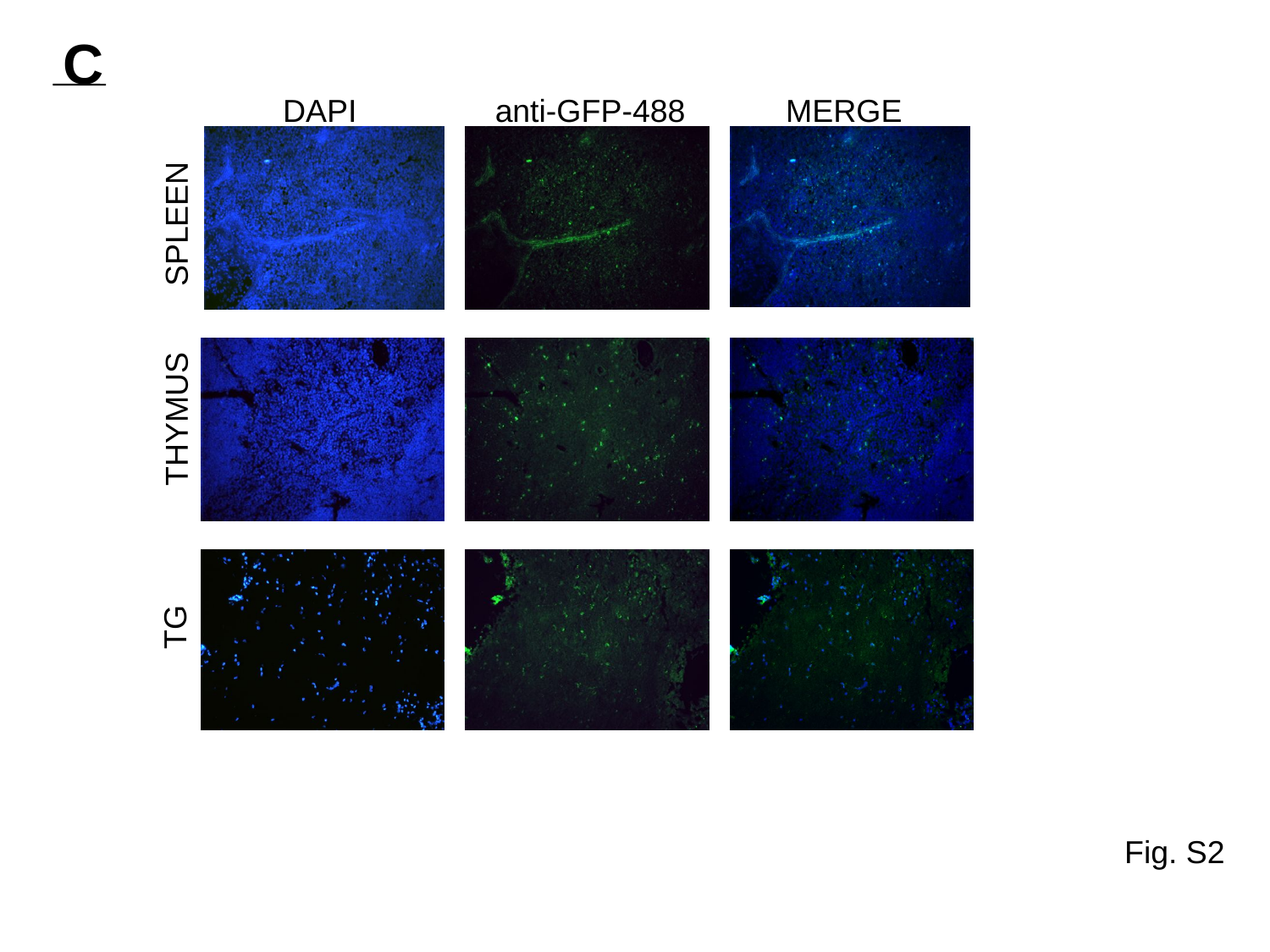

C
DAPI
anti-GFP-488
MERGE
SPLEEN
THYMUS
TG
Fig. S2
