# Supplementary material for: CD8α Dendritic Cells Drive Establishment of HSV-1 Latency
Source: PLoS One. 2014 Apr 2;9(4):e93444. doi: 10.1371/journal.pone.0093444 (PMC3973679; doi:10.1371/journal.pone.0093444)
Supplement: Table S1 — Survival of different mouse strains following ocular infection. (DOCX) [file pone.0093444.s002.docx]

**Table S1. Survival of different mouse strains following ocular infection.a**

| PFU/eye of McKrae | | | PFU/eye of KOS |  |
| --- | --- | --- | --- | --- |
| Mouse Genotype | 2x10^5^ | 2x10^4^ | 2x10^3^ | 2x10^5^ |
| C57BL/6J | 19/20 (95%) | ND | ND | ND |
| β2m-/- | 9/10 (90%) | ND | ND | ND |
| CD8α^-/-^ | 9/10 (90%) | ND | ND | ND |
| CD8β^-/-^ | 12/12 (100%) | ND | ND | ND |
| BXH2/TyJ | 0/10 (0%) | 1/5 (20%) | 9/10 (90%) | 5/5 (100%) |
| C3H/HEJ | 0/10 (0%) | 0/5 (0%) | 6/10 (60%) | 5/5 (100%) |

aMice were infected ocularly with different PFU of McKrae without corneal scarification. Ocularly infected mice were monitored daily for 28 days including weekends and holidays. Infected mice that were not able to access food and water were euthanized by CO_2_ inhalation followed by cervical dislocation. On day 28 post infection all surviving mice were euthanized by CO_2_ inhalation followed by cervical dislocation. ND = Not Done.
